# Nutrient Composition of Foods Marketed to Children or Adolescents Sold in the Spanish Market: Are They Any Better?

**DOI:** 10.3390/ijerph17207699

**Published:** 2020-10-21

**Authors:** Marta Beltrá, Keila Soares-Micoanski, Eva-Maria Navarrete-Muñoz, Ana B. Ropero

**Affiliations:** 1 Institute of Bioengineering, Miguel Hernández University, 03202 Elche, Spain; keilamicoanski@outlook.com (K.S.-M.); ropero@umh.es (A.B.R.); 2 Grupo de Investigación en Terapia Ocupacional (InTeO), Department of Surgery and Pathology, Miguel Hernández University, 03550 Alicante, Spain; enavarrete@umh.es

**Keywords:** children, adolescents, nutrient composition, healthy, macronutrients, marketing

## Abstract

Healthy eating is essential for the growth and development of children and adolescents. Eating habits established in childhood continue into adulthood. In Spain, the frequent promotion of foods with low nutritional value is already considered a threat to the health of the population, particularly to children and adolescents. In this work, we analyse 3209 foods from the Food Database, BADALI. Foods were classified as marketed to children or adolescents according to the advertising on the packaging, television or internet. We found that 17.5% of foods in the database were marketed to this population and 97% of those were considered unhealthy following the Pan American Health Organization Nutrient Profile Model (PAHO-NPM). In the total of foods for children or adolescents, 61.5% were high in fat, 58.5% in free-sugar, 45.4% in saturated fat and 45% in sodium. Foods marketed to them presented higher amounts of carbohydrates and sugar, while lower protein and fibre content than the rest. There was also considerable variability in levels of the other nutrients found in these products, which depended largely on the food group. According to our findings, there is a tendency for products marketed to children or adolescents to be unhealthy and of a poorer nutritional quality than those not targeted at them.

## 1. Introduction

Overweight and obese children are at higher risk of developing serious health problems, including type 2 diabetes, high blood pressure, asthma and other respiratory problems, sleep disorders and liver disease [1]. They may also suffer from psychological effects, such as low self-esteem, depression and social isolation [1]. Childhood obesity also increases the risk of Non-Communicable Diseases (NCDs), premature death and disability in adulthood [1]. According to the World Health Organization (WHO), the countries of southern Europe, including Spain, have the highest rates of childhood obesity in Europe (18–21%) [2]. In Spain, the prevalence of overweight and obesity was 54.5% in adults and 28.6% in children and adolescents in 2018 [3,4]. Incorrect eating habits, excess energy, imbalance in macronutrient quality and micronutrient deficiency are associated with metabolic changes, increased risk of obesity and NCDs [5,6,7].

Healthy eating is essential for the growth and development of children and adolescents. Eating habits established during childhood continue into adulthood [2]. Although energy balance is important in the short term for weight control, the eating pattern is the determining factor in the long term [3,4]. The Vienna Declaration on Nutrition and Noncommunicable Diseases in the Context of Health 2020, issued by the WHO in 2013, acknowledges that a “healthy diet can contribute to achieving the global targets on NCDs” [8]. It also reaffirms the commitment to existing European and global frameworks to address important NCDs risk factors, notably unhealthy diet and physical inactivity, pursuing a 25% relative reduction in premature mortality from NCDs by 2025 [9].

According to the Spanish Federation of Food and Beverage Industries annual report in 2018, the food and beverages industries are the main industrial sector in the country [10]. Markets offer a wide variety of foods and beverages, while advertising has a far wider reach and focuses mainly on products with high levels of fat, sugar and/or salt [1]. The marketing of these products has been acknowledged in Europe as one of the risk factors contributing to childhood obesity and the development of NCDs [11]. Globally, there has been an increased intake of energy-dense foods that are high in fat and sugar [8].

In 2013, the WHO already stated, “Unfortunately, marketing unhealthy food to children has been proven to be disastrously effective. While adults know when they are being targeted by advertising, children cannot distinguish, for example, between advertisements and cartoons. This makes them particularly receptive and vulnerable to messages that lead to unhealthy choices” [12]. The leading groups of advertised foods are soft drinks, sweetened breakfast cereals, biscuits, confectionery, snack foods, ready meals and fast food restaurants [13]. In Spain, the frequent promotion of foods with low nutritional value is already considered a threat to the health of the population. This is particularly worrying because many of these products make nutrition and health claims and many of them are aimed at children [6,12]. According to the National Survey of Nutrition in the Child and Adolescent Population (ENALIA) in 6 to 9 year old children, the intake of products high in fat, sugar and/or salt is a serious problem. Of all the children surveyed, 70.4% were pastry consumers, with an average of 64.8 g/day, with 256 g/day of juice for consumers, dairy desserts at 157 g/day, breakfast cereals at 34.5 g/day and soft drinks at 253 g/day [14]. In view of this worldwide situation, the WHO Child Obesity Surveillance Initiative recommends limiting the intake of savoury snacks, fast foods, processed meat and sugary soft drinks. Instead, they promote eating fruits and vegetables on a daily basis [15].

Previous studies have shown that most foods marketed to children are considered unhealthy or “less healthy” regardless of the country of study [16,17,18,19,20,21,22]. High levels of sugar, saturated fat, sodium and total fat were observed in a great proportion of these foods [18,19,20,21,22]. In Spain, the situation is not better. In a paper published in 2012, the authors found that 61% of products advertised during children’s viewing time were “less healthy” [23]. Campos et al. reported in 2016 that 46–57% of food advertisements on TV during children’s peak time slots were on foods high in energy or with an unbalanced energy profile [24].

Only a few papers address the nutritional differences between foods marketed to children and those not marketed to them [22,25,26,27]. A study in the USA found that the increased presence of child-targeted cues was associated with more sugar and less fibre and protein [27]. To our knowledge, to date, no similar publications have been found for the Spanish market.

There is limited information about the nutritional quality of foods marketed to children or adolescents in Spain. In addition, no publications have been found comparing them with foods not targeted at this population in our country.

Therefore, the aim of this work was to evaluate the nutrient composition of foods marketed to children or adolescents in the Spanish market and to compare them with those not targeted at them.

## 2. Materials and Methods

### 2.1. Database of Food Products Available in the Spanish Market

The information used in this work comes from the Food Database, BADALI, freely available online [28,29]. Details about the food and brand selection process can be consulted in Ropero et al. 2020 [30]. 3209 foods present in the Spanish market were collected from August 2013 to August 2020, belonging to 162 different brands. The food information used in this study was obtained from the manufacturers’ webpages, including the nutrient composition and images of the packaging.

Nutrient composition of foods was extracted by the researchers and inconsistent information was not used for further analysis. The following information for each food product was collected: brand name, name, energy (kcal/100 g), protein (g/100 g), carbohydrate (g/100 g), starch (g/100 g), sugars (g/100 g), total fat (g/100 g), monounsaturated fats (g/100 g), polyunsaturated fats (g/100 g), saturated fats (g/100 g), cholesterol (mg/100 g), ω 3 fatty acids (g/100 g), ω 6 fatty acids (mg/100 g), fibre (g/100 g) and salt (g/100 g).

### 2.2. Food Classification into Study Categories

Two independent researchers examined all the foods in the database in order to identify those marketed to children or adolescents (Ch/Ad category). Discrepancies between researchers were resolved by consulting a third author. Foods were included in the Ch/Ad category when the product satisfied at least one of the following criteria: (1) the product packaging featured an image of licensed characters, a television theme or a film related to children or adolescents; (2) the packaging contained any other cartoon design; (3) the packaging contained an activity or promotion targeted at children or adolescents (for example, a toy); (4) presence of food advertising on TV or Internet targeted at children or adolescents; (5) the product was listed as “kids food” on the manufacturer’s website; (6) the brand was considered a secondary brand (one imitating the original) of an original product already included in this group; or (7) food shape represented a cartoon, or any design related to children or adolescents, such as pasta in letter format or following children’s drawings.

The remainder of the foods were classified in the “non-Ch/Ad” category. Foods in the database were grouped into 25 food groups (Table 1). Only those groups with at least 15 foods for both categories were analysed. Likewise, individual nutrients were studied as long as the sample size was 15 or more.

### 2.3. Classification of Products According to Their Nutrient Profile

The Pan American Health Organization Nutrient Profile Model (PAHO-NPM) was used to classify foods as healthy or unhealthy following previous works [20,31,32,33]. It provides thresholds for detecting foods high in critical nutrients, i.e., sodium, free sugar, total, saturated and trans-fat. It also considers the presence of other sweeteners in the product. In this work, foods were considered unhealthy when they were high in sodium, free sugar, total or saturated fat (the database does not include values for trans-fat). Thresholds used were as follows: (1) ≥1 mg sodium/kcal, (2) ≥10% of total energy from free sugars, (3) ≥30% of total energy from total fat and (4) ≥10% of total energy from saturated fat [33].

Free sugars were estimated based on the method included in the PAHO-NPM [33]. However, some extra criteria were used: (1) for vegetables, fruits, dairies, legumes, cereals, nuts and seeds with no added sugar, the % of energy as free sugar was 0; (2) for derivatives of vegetables, fruits, legumes, cereals, chocolates, nuts and seeds with added sugar, the amount of naturally occurring sugar in the original natural food was obtained from the Spanish Food Composition Database (BEDCA) or BADALI [28,34] and subtracted (cocoa for chocolates); (3) 5 g sugar/100 g was subtracted from total sugar for all dairy products, since this is the naturally occurring lactose content in milk [34]; (4) all the sugar in fruit beverages and soft drinks is free sugar; (5) since meat, fish/seafood and sweets do not contain appreciable amounts of naturally occurring sugar, total sugar was considered free and (6) for sauces, the naturally occurring sugar was estimated from the ingredients.

The PAHO-NPM was not intended for use with unprocessed or minimally processed foods. Therefore, we checked for this type of foods in the Ch/Ad category in the BADALI database and 32 products met the criteria. Three were unsweetened yoghurts and three were sunflower seeds with no added ingredients. We considered them healthy. The rest were juices. Since the definition of free sugar by the WHO includes those in juices, we classified them as unhealthy [35].

### 2.4. Data Analysis

Foods’ nutrient compositions were included in spreadsheets of Microsoft Office 365, Excel^®^. All statistical analysis was performed using the R software, version R 4.0.0 (R Core Team. R: A language and environment for statistical computing. R Foundation for Statistical Computing, Vienna, Austria; http://www.R-project.org). All applied statistical tests were bilateral and significance level was established at 0.05.

To check whether continuous variables were normally distributed, we used the Kolmogorov–Smirnov test. The nutrient composition was described using the median and interquartile range to reduce skewness and optimize the normality of the distribution of the variables. The Mann–Whitney test for continuous variables was applied to examine differences in the nutrient composition according to the two categories of study.

## 3. Results

### 3.1. Data Description and General Overview

Within the complete food sample, the five most numerous food groups were Groups (G) 1, 7, 13, 16 and 25 (Table 2). Of the 3209 products included in the database, 563 of them (17.5%) were classified into the children or adolescents category (Ch/Ad) and 2646 (82.5%) were grouped into the non-children or adolescents category (non-Ch/Ad) (Table 2). According to these two categories, some differences were observed. For Ch/Ad, G1, G7, G9, G23 and G25 were the most numerous. As for non-Ch/Ad, G1, G13, G16, G21 and G25 were the most prevalent (Table 2).

When food groups were analysed individually, as much as 60% of Snacks (G23) were marketed to children or adolescents. Six more groups had at least 30% of foods classified into that category (G1, G2, G12, G19, G20 and G24). On the contrary, none of the foods in G4, G8, G10, G11 and G18 were in the Ch/Ad category. In addition, six groups had less than 10 foods (G2, G5, G13, G15, G17 and G21).

### 3.2. Healthiness of Foods Marketed to Children or Adolescents

As many as 97% of all foods in the Ch/Ad category were high in at least one nutrient and consequently, were considered unhealthy (Table 3). In the total of foods, 61.5% of them were high in fat, 58.5% were high in free sugar, 45.4% were high in saturated fat and 45% were high in sodium. All groups had a prevalence of unhealthy foods higher than 90%. When individual nutrients were analysed by groups, the distribution of foods high in critical nutrients was found to be heterogeneous. Four groups presented more than 50% of foods high in 3 of the 4 nutrients analysed (G1, G6, G16 and G19). Five groups had more than 50% of foods high in 2 nutrients (G7, G14, G20, G23 and G25).

### 3.3. Nutritional Differences Between Foods Marketed or Not to Children or Adolescents

Table 4 shows the nutrient composition of all the foods included in the BADALI database by categories. The most striking difference was for total fat, with a 4-fold increase in the Ch/Ad category. In addition, they presented higher content of energy, carbohydrate, sugars, salt, monounsaturated and saturated fats. At the same time, they had lower content of protein and fibre.

Since the differences observed in the entire sample may be the consequence of the heterogeneous distribution of foods into the two categories, food groups were analysed individually (Table 5 and Table 6). The nutrient composition of foods in the two categories was different for all nutrients, except for polyunsaturated fats. Dissimilarities in carbohydrate and sugar were the result of higher content in 7 and 8 food groups respectively for the Ch/Ad (Table 5). It is worth mentioning that sugar content in beverages is higher for Ch/Ad. Regarding fibre, results showed lower values for cereal subgroups (G1 and G19). For the rest of the nutrients, variable results were observed depending on the food group.

All of the food groups presented statistically significant differences in at least two nutrients. G1, G6, G12 and G25 had differences in 6–7 nutrients of the 8 analysed (Table 5 and Table 6). Cheese (G6) marketed to children or adolescents had a better nutritional profile with lower energy, salt, total and saturated fat values. This is because fresh cheese was preferably classified into the Ch/Ad category (19 of 21), while mature cheese in the non-Ch/Ad (76 of 81) (Table 5 and Table 6).

Biscuits, breakfast cereals and cereal bars (G1) marketed to children or adolescents had lower nutritional quality: more energy-dense, sugar, total and saturated fat content, while lower in protein and fibre (Table 5 and Table 6). Some of these differences may be due to a higher presence of chocolate in the Ch/Ad category. In fact, 62% of all foods in this group had chocolate, while the equivalent for the non-Ch/Ad category was 21%. Milk and dairy beverages showed similar results. Energy, carbohydrates and sugars were higher for Ch/Ad because 15 of the 16 beverages with added sugar were classified into this category. As for processed nuts and seeds, those for Ch/Ad had 4-fold salt content because of salty sunflower seeds (all 15 in this category).

The differences observed in sweets and chocolates were due to the classification of most candies into the Ch/Ad category (20 of 24), while all of the 45 high-cocoa chocolates were in the non-Ch/Ad (cocoa content ≥ 50%). As for sauces, the striking increases in energy and total fat levels in the non-Ch/Ad category were because 32 of the 34 fat-based sauces were included here (fat ≥ 30%).

## 4. Discussion

Our study provided an overview of the nutritional quality of Spanish foods marketed to children or adolescents (Ch/Ad). It also compared them with those foods not targeted at this population. We found that 17.5% of foods analysed were classified into the Ch/Ad category. The distribution of these foods among groups was heterogeneous. Ninety-seven percent of them were high in at least one of the critical nutrients according to the PAHO-NPM and, therefore, considered unhealthy. All groups had a prevalence of unhealthy foods above 90%. In the total of foods for children or adolescents, 61.5% of them were high in fat, 58.5% in free sugar, 45.4% in saturated fat and 45% in sodium. The distribution of foods high in critical nutrients was heterogeneous among groups. When the nutrient composition was compared, those marketed to children or adolescents showed a poorer nutritional quality than the rest. A four-fold increase in fat and higher energy, carbohydrate, sugar, salt and saturated fats content was observed for the Ch/Ad category, while protein and fibre values were lower. The dissimilarities in carbohydrates, sugar and fibre were quite homogeneous throughout food groups, albeit there was variability depending on the food group for the rest of the nutrients.

Results in the present work showed a similar proportion of foods targeted at children or adolescents (17.5%) to the ones obtained in a large study in the USA evaluating 56,900 total foods (9105 products, 16%) [19]. Lower rates were observed in a work carried out in Brazil with 5620 packaged foods (9.5%) and in another in Slovenia (5.3% of 8191 total prepacked foods) [16,25]. Two publications on breakfast cereals showed variable proportions of foods targeted at children: 46% in a sample from the USA, while 17% in another from supermarkets in Auckland, New Zealand [22,36]. Our data on biscuits, breakfast cereals and cereal bars rendered an intermediate value of 33.1%. Regarding yoghurts, a study in the UK found that 41% were targeted at children, while only 22.9% in our case [26]. Differences may be because other fermented milk and dairy desserts were also included in this group.

### 4.1. Are Foods Marketed to Children or Adolescents Healthy?

The application of the PAHO-NPM to foods targeted at children or adolescents in the BADALI database rendered as much as 97% of unhealthy foods. A similar value was obtained in a work carried out in Slovenia (93%) [16]. Lower rates were reported in other publications. Applying the UK/Ofcom nutrient profile model, 75% of packaged foods targeted at children in Brazil were found to be “less healthy” [17]. In a study in Spain published in 2012, the authors found that 61% of the 96 foods advertised on TV during Children’s viewing time were “less healthy” [23]. All foods in the biscuits, breakfast cereals and cereal bars in our study were unhealthy. However, in a study in New Zealand, 58% of breakfast cereals for kids were considered “less healthy” by using the nutrient profiling tool developed by the Food Standards Australia New Zealand [22].

One of the aims of the PAHO-NPM was to be used as a tool to restrict the marketing of unhealthy foods and beverages to children [33]. Several other institutions have developed nutrient profiling models over the last two decades, with the WHO’s European Regional Office (WHOE) [37], the WHO’s Eastern Mediterranean Regional Office [38] and the UK Food Standards Agency [39] among them. Only foods complying with the criteria could be advertised. According to the present work, establishing such a system to prevent the marketing of unhealthy foods in Europe would mean that 97% of the present promotion of foods to children and adolescents would not be permitted. In fact, the European Commission proposed to develop a nutrient profile model in 2006, but it has never been established [40].

Our data shows that 61.5% of products marketed to children or adolescents were high in fat, 58.5% in free sugar, 45.4% in saturated fat and 45% in sodium. These results can be compared to previous studies. In a work carried out in Uruguay with 180 foods aimed at children, 91% of them had an excessive amount of free sugar, 50% of saturated fat and 40% of total fat according to the same PAHO-NPM [20]. In a large study in the USA (9105 foods marketed to children), 63% of all products had a high content of saturated fat, sodium and/or sugar [19]. Several studies analysed foods targeted at children in Canada. In one, 89% of the 367 foods analysed could be classified as of poor nutritional quality: 69.5% had high levels of sugar, 22.7% of total fat and 17% of sodium [41]. High levels of sugar and/or sodium were also observed in 63% of 186 foods for babies and toddlers [21]. Another work found that 77.8% of foods had excess free sugars (747 total foods) [18]. In Spain, a study published in 2016 obtained that 46–57% of food advertisements on TV during children’s peak time slots were on foods high in energy or had an unbalanced energy profile [24].

Therefore, foods marketed to children or adolescents can be considered of poor quality or “less healthy”. In fact, our data shows that several groups with only a few foods or none targeted at this population were of good nutritional quality according to the description of each group in Table 1. This is the case for legumes, natural/toasted nuts, fresh and frozen fish and seafood, rice, cereal pasta, germ, bran, flours and other processed cereals and bread (except for the salt added). In this line, findings from a Canadian study showed that only 1% of foods marketed to children were represented by fruits and vegetables [41].

### 4.2. Are Foods Marketed to Children or Adolescents Healthier Than the Ones Not Targeted at This Population?

Carbohydrates, sugar, fibre and protein showed important and consistent differences in foods for children or adolescents in the present work. This is in agreement with a study in the USA with 715 foods. Authors found that the increased presence of child-targeted cues was associated with more sugar and less fibre and protein [27].

In a study in Brazil, authors audited 5620 packaged foods and found variable results, depending on the food group [25]. They found higher carbohydrates content for children’s foods in three groups. They did not analyse sugar content. Regarding fibre, they showed lower content in the two main groups with vegetable-derived foods. Saturated fats were lower for children’s food in three groups, while increased in one. Our data show a similar tendency to a lower content of this nutrient in some groups. As for sodium, half of the groups had consistently lower levels for children, which was not the case in our study [23]. Results from this study and ours can hardly be compared. The main reason is that they classified foods in groups following different criteria. Baking goods, bread, cereals, legumes, roots and tubers are all in the same food group. However, in our study, we classified them separately. Similar differences were observed for other food groups.

A publication on breakfast cereals confirmed that those marketed to children had more energy, sugar and sodium, while less fibre and protein [36]. Except for the salt, these results were similar to ours, considering that we included biscuits and cereal bars in the same food group. Another study in New Zealand obtained that more cereals for kids were considered “less healthy” than the rest [22]. When yoghurts were analysed in a sample in the UK, the ones marketed to children contained higher amounts of sugars, energy, total and saturated fat than their non-children counterparts [26]. We did not obtain such differences, most probably because some fermented milk and dairy desserts were included in the same food group.

### 4.3. Consequences of Unhealthy Foods for Children or Adolescents

The most consistent difference between foods marketed to children or adolescents and the rest of foods in the present work was with sugar. According to the WHO, free sugar intake is associated with poor dietary quality, obesity and risk of NCDs [35]. In fact, in a study in American preschoolers, increasing added sugar consumption was associated with increased proportions of children with intakes below the dietary reference intakes for some nutrients [42].

The free sugar intake in the Spanish children and adolescent population exceeds the WHO recommendations of lower than 5% of the daily energy [43]. A calculation from the “Anthropometry, Intake, and Energy Balance in Spain” study (ANIBES) rendered at least 11% of daily energy intake as free sugars in the population aged between 9 and 17 years [43]. Therefore, an important reduction in free sugar intake is highly advisable in this population.

Fibre content is lower in foods aimed at children or adolescents in the two groups derived from cereals. In fact, the medians and the interquartile ranges correspond to products prepared with refined cereals [34]. According to the ENALIA study, an important proportion of children and adolescents do not follow the European Food Safety Authority (EFSA) recommendations for fibre [14,44,45]. Results of the National Health and Nutrition Examination Survey (NHANES study) indicated that the increase in dietary fibre intake is related to a lower risk of childhood obesity [46]. A diet rich in fibre and bran improves constipation and it is recommended to children with symptoms [47]. Another important reason to increase the intake of wholegrain cereals is the higher content of vitamins and minerals [48]. The consequence of refining whole wheat, the main cereal in Spain, is the loss of large amounts of vitamin B, calcium, iron, potassium, magnesium, phosphorus, selenium and zinc [45]. In fact, children and adolescents have some degree of deficit in folic acid, calcium, iron, magnesium and potassium [46,48]. Besides, the consumption of wholegrain foods may provide several benefits for human health. Wholegrains intake has been correlated with a reduction of weight gain and the risk of obesity, type 2 diabetes and cardiovascular disease [49]. The World Cancer Research Foundation, in the 2018-Continuous Update Project (CUP) report, concluded that there is strong evidence that consuming wholegrains helps protecting against colorectal cancer [50]. Therefore, moving from refined to wholegrain cereals may greatly improve children and adolescents’ diets on micronutrients and fibre, as well as having direct effects on their health.

Our results showed that 61.5% of all foods marketed to children or adolescents were high in total fat. In Spain, the fat intake in this population exceeds 38% of the daily energy intake [51]. Diets high in total fat can be beneficial to health as long as most of them are unsaturated fats [52,53]. This is not the case in the Spanish children and adolescents population [45].

In the present work, 46% of all foods marketed to children or adolescents were high in saturated fat. Excessive saturated and trans-fat content in the diet increases morbidity and mortality from cardiovascular diseases in adults and children [54]. According to EFSA, the main dietary determinants of blood LDL-cholesterol concentrations are saturated and trans-fat intake [55]. Results from the ENALIA study show that the intake of saturated fat is above recommendations in more than 89% of Spanish children and adolescents [45]. Therefore, a reduction in this nutrient is strongly advisable.

Forty-five percent of foods for children or adolescents in our study were high in sodium. Excessive sodium consumption contributes to a series of adverse health outcomes, as it is an adjunctive food risk factor for hypertension, cardiovascular disease and death [56,57,58]. In fact, the “WHO Global Action Plan for the Prevention and Control of NCDs 2013–2020” aims at reducing the average sodium intake in the population by 30% [59]. Therefore, a reduction in sodium intake for all citizens is most desirable to improve their health status.

According to ANIBES, 76.9% of Spanish children and adolescents do not follow a healthy lifestyle regarding eating and physical activity [60]. The “Health Behaviour in School-aged Children” (HBSC) study performed in 2018 showed that only 34.7% and 27.1% of adolescents eat fruit and vegetables daily, respectively [61]. At the same time, 72.8% eat sweets and 65.5% consume soft drinks and sugary beverages at least once a week [61]. The ENALIA study also showed low levels of fruit, vegetables and legumes intake in children and adolescents, while they ate more than 46 g/day of pastries and more than 27 g/day of processed meat [14,45]. They also had more than 90 g/day of sugary soft drinks, except for girls following a healthy lifestyle [14,45]. Pastries, soft drinks and sweets and chocolates are groups with a high proportion of foods targeted at children or adolescents in the present study, while only a few legumes were marketed to this population.

### 4.4. Strengths and Limitations of the Study

This study has a few strengths worth mentioning: (1) more than 3000 foods were analysed, which falls within the average sample size of other publications; (2) data was collected following criteria completely unrelated to the aim of this study or the targeted population and, as a consequence, our results lack any bias on food choice; (3) foods from all groups were analysed, which provided an overview of products in the Spanish market and (4) the requirement for having a minimum of 15 foods from each food group and for the analysis of individual nutrients.

At the same time, we are well aware of the limitations: (1) data collected was reliant on the accuracy of the information provided in the manufacturer’s webpage; (2) selection of brands did not follow a criteria based on customers’ purchase or most popular products; (3) the 3209 foods analysed may not be fully representative due to the huge amount of foods available in the market; (4) fresh foods were not analysed because they are exempted from the requirement of the mandatory nutrition declaration [62] and, therefore, were not included in the database; (5) there may have been changes in nutrient composition since the information was collected and (6) there is not a standardised protocol for classifying foods marketed to children or adolescents, and consequently, some foods may not have been placed in their most appropriate category.

## 5. Conclusions

The present study showed that nearly all of the 563 analysed foods marketed to children or adolescents in the Spanish market were found to be unhealthy (high in fat, free sugar, saturated fat or sodium). When they were compared to foods not targeted at this population, they were of inferior nutritional quality (higher energy, sugar, salt, total and saturated fat levels combined with reduced protein and fibre content).

In recent years, institutions have attempted to develop programs to address the high rates of overweight and obesity in children. To date, these programs have not been successful. To improve children and adolescents’ food habits, every party involved should contribute to this: governments, social services, educational institutions, families and industries. As it is the case in some countries, governments should limit the marketing of unhealthy foods for children. Families should prioritise fruits, vegetables, legumes, wholegrains and nuts. Industries should be truly involved in the reformulation of foods to make them healthier by reducing the fat, sugar, energy and salt content of processed foods. Only with this type of compromise will the food habits of our children and adolescents be improved and, hopefully, help them to become part of a future healthier adult population.

## Figures and Tables

**Table 1:** Description of the items included in the food groups.

Food Group	Foods
G1—Biscuits, breakfast cereals and cereal bars	Puffed rice, oatmeal, flakes, bran, filled biscuits, wholegrain products, regular biscuits, biscuits with chocolate
G2—Bread and sliced bread	Bread (normal, sliced, hamburger, doggy), wholegrain
G3—Canned, processed vegetables and derivatives	Creams and soups, pickled gherkins, gazpacho, tomato-based sauces, vegetable jams, salads, canned and processed vegetables, sweet corn
G4—Cereal cakes and toasts	Cakes (rice, wheat, corn, oat, spelt), cereal toasts
G5—Cereal derivatives	Snacks, pizza base, doughs, nachos, wheat sticks, dehydrated soup
G6—Cheeses	All types of cheeses (fresh, cured or semi-cured, melted, with spices), mousse cheese
G7—Derivatives and processed fish/seafood	Tuna pate, surimi, canned and smoked fish and seafood
G8—Fats	Margarines, lard
G9—Fermented milk and dairy desserts	Yogurts, other fermented milk. Desserts (custard, flan, catalane creams), chocolate and other flavoured mousses, rice pudding
G10—Fresh and frozen fish/seafood	Fresh, chilled or frozen
G11—Fruits	Dried fruit, olives, olive pates, canned fruit, candied fruit, jams, fruit spreads, sweet quinces
G12—Fruit beverages and juices	Nectars, fruit beverages, musts, horchatas, juices
G13—Legumes	Dried, canned, fermented soy, soy desserts, textured soybeans, humus, legumes pasta and flour, chips, other derivatives
G14—Milk and dairy beverages	Milk (liquid, powder, condensed or evaporated), milk shakes, milk with other ingredients
G15—Milk derivatives	Butter, cream, spreading creams with cheese
G16—Meat	Cold meat, luncheon meat, pate
G17—Natural or toasted nuts and seeds	Unprocessed or toasted nuts and seeds (pumpkin, poppies, sunflower, flax, chia, hemp)
G18—Other beverages	Non-alcoholic beers, vegetable beverages
G19—Pastries and cakes	Donuts, muffins, gluten-free cupcake, croissants, profiteroles, ready-to-use mixes for pastries
G20—Processed nuts and seeds	Salted nuts
G21—Rice, cereal pasta, germ, bran, flour, natural/toasted and other processed cereals	Rice, brown rice, cereal pasta (wholegrain and refined), breadcrumbs, doughs, flour (wheat, rice, rye, corn, wholegrain), bran (oats, wheat, spelled), couscous, bulgur, amaranth, quinoa, oats, barley, rye, millet, wheat, corn, spelt
G22—Sauces	Ketchup, mayonnaises, other sauces
G23—Snacks	Potato chips, processed potatoes, popcorn, corn snacks
G24—Soft drinks	Cola, orange and lemon sodas, flavoured water with sweeteners, tonics
G25—Sweets and chocolates	Chewing gums, caramels, wafers, honey, candies, syrups, sweet creams, chocolate (bars, filled and powder), chocolate-coated cereal bars

**Table 2:** Items included in each food group in the total of the Food Database, BADALI, and classified into categories (Ch/Ad and non-Ch/Ad).

Food Groups	Total (%)	Ch/Ad (%)	Non-Ch/Ad (%)
G1—Biscuits, breakfast cereals and cereal bars	317 (9.9)	105 (18.7)	212 (8)
G2—Bread and sliced bread	28 (0.9)	9 (1.6)	19 (0.7)
G3—Canned, processed vegetables and derivatives	185 (5.7)	25 (4.4)	160 (6)
G4—Cereal cakes and toasts	58 (1.8)	0 (0)	58 (2.2)
G5—Cereal derivatives	54 (1.7)	2 (0.4)	52 (2)
G6—Cheeses	164 (5.1)	33 (5.9)	131 (5)
G7—Derivatives and processed fish/seafood	256 (8)	52 (9.2)	204 (7.7)
G8—Fats	13 (0.4)	0 (0)	13 (0.5)
G9—Fermented milk and dairy desserts	153 (4.8)	35 (6.17)	118 (4.5)
G10—Fresh and frozen fish/seafood	55 (1.7)	0 (0)	55 (2.1)
G11—Fruits	172 (5.4)	0 (0)	172 (6.5)
G12—Fruit beverages and juices	107 (3.3)	33 (6.2)	74 (2.8)
G13—Legumes	254 (7.9)	7 (1.2)	247 (9.3)
G14—Milk and dairy beverages	86 (2.7)	16 (2.8)	70 (2.6)
G15—Milk derivatives	63 (2)	5 (0.9)	58 (2.2)
G16—Meat	262 (8.2)	28 (5)	234 (8.8)
G17—Natural or toasted nuts and seeds	45 (1.4)	3 (0.5)	42 (1.6)
G18—Other beverages	68 (2.1)	0 (0)	68 (2.6)
G19—Pastries and cakes	75 (2.3)	27 (4.8)	48 (1.8)
G20—Processed nuts and seeds	48 (1.6)	15 (2.7)	33 (1.3)
G21—Rice, cereal pasta, germ, bran, flour, natural/toasted and other processed cereals	214 (6.7)	4 (0.7)	210 (7.9)
G22—Sauces	84 (2.6)	15 (2.7)	69 (2.6)
G23—Snacks	93 (3)	56 (10)	37 (1.4)
G24—Soft drinks	85 (2.6)	28 (5)	57 (2.2)
G25—Sweets and chocolates	271 (8.4)	66 (11.7)	205 (7.8)
Total	3209 (100)	563 (17.5)	2646 (82.5)

**Table 3:** Classification of foods marketed to children or adolescents (Ch/Ad category) as healthy/unhealthy according to the Pan American Health Organization Nutrient Profile Model (PAHO-NPM) *.

Food Groups	High in Any Nutrient		High Fat		High Free-Sugar		High Saturated Fat		High Sodium	
	n	No (%)	n	No (%)	n	No (%)	n	No (%)	n	No (%)
G1—Biscuits, breakfast cereals and cereal bars	104	104 (100)	102	85 (83.3)	104	98 (94.2)	102	61 (59.8)	103	20 (17.4)
G3—Canned, processed vegetables and derivatives	25	23 (92)	25	10 (40)	25	9 (36)	25	7 (28)	25	23 (92)
G6—Cheese	33	32 (97)	33	32 (97)	33	0 (0)	33	32 (97)	32	29 (87.9)
G7—Derivatives and processed fish/seafood	52	51 (98.1)	52	36 (69.3)	49	0 (0)	52	10 (19.23)	51	47 (92.2)
G9—Fermented milk and dairy desserts	32	29 (90.6)	32	8 (25)	31	11 (35.5)	31	26 (83.9)	31	0 (0)
G12—Fruit beverages and juices	33	33 (100)	33	0(0)	33	33 (100)	32	0 (0)	33	0 (0)
G14—Milk and dairy beverages	16	16 (100)	16	0 (0)	16	15 (93.8)	16	12 (81.3)	16	7 (37.5)
G16—Meat	28	28 (100)	28	27 (96.4)	20	0 (0)	20	18 (90)	28	28 (100)
G19—Pastries and cakes	28	28 (100)	28	26 (92.8)	25	24 (96)	24	17 (70.8)	26	2 (7.7)
G20—Processed nuts and seeds	15	15 (100)	15	15 (100)	15	0 (0)	15	0 (0)	15	10 (66.7)
G22—Sauces	15	15 (100)	15	4 (26.7)	11	8 (ND)	15	3 (20)	8	8 (ND)
G23—Snacks	55	54 (98.2)	55	51 (92.7)	55	4 (7.3)	55	20 (36.4)	53	36 (67.9)
G24—Soft drinks	28	27 (96.4)	28	0 (0)	28	27 (96.4)	28	0(0)	28	12 (42.9)
G25—Sweets and chocolates	67	67 (100)	67	43 (64.2)	51	50 (98)	51	22 (45.1)	51	1 (2)
Total	563	547 (97.1)	563	346 (61.5)	515	301 (58.5)	522	237 (45.4)	533	240 (45)

* Thresholds used to consider foods as high in critical nutrients [36]: ≥30% of total energy from total fat, ≥10% of total energy from free sugars, ≥10% of total energy from saturated fat, ≥1 mg sodium/kcal.

**Table 4:** Nutrient composition of all the foods included in the database according to category of study.

Nutrient Content for 100 g of The Product	Ch/Ad		Non-Ch/Ad		*p*-Value
	N	Median (IR)	n	Median (IR)	
Energy (Kcal)	563	308 (98; 491)	2634	272 (90; 398)	<0.001
Protein(g)	560	5.8 (3; 10)	2638	7.0 (2.9; 15)	<0.001
Carbohydrates (g)	552	23.9 (6.1; 63)	2591	13.6 (3.9; 56)	<0.001
Starch (g)	22	11.4 (0.2; 29)	31	42.9 (32; 52)	<0.001
Sugars (g)	513	9.65 (2.3; 28)	2293	3.5 (1; 12.8)	<0.001
Total fat (g)	563	15 (1.9; 24)	2637	3.6 (1; 20)	<0.001
Monounsaturated fats (g)	92	8 (1; 12)	283	2.5 (0.5; 11)	0.013
Polyunsaturated fats (g)	94	2.0 (1; 2)	290	1.5 (1; 2.4)	0.988
Saturated fats (g)	522	2.9 (0.7; 7.7)	2301	1.3 (0.1; 5.8)	<0.001
Cholesterol (mg)	7	26 (9; 30)	36	60 (36.3; 156.5)	ND
ω 3 fatty acids (g)	10	0.3 (0.3; 0.3)	51	1 (0.2; 6.3)	ND
ω 6 fatty acids (mg)	0	--	19	15 (0.1; 23)	ND
Fibre (g)	277	2.3 (1; 3.5)	1456	3 (1.1; 6.2)	<0.001
Salt (g)	533	0.6 (0.1; 1.3)	2390	0.25 (0.05; 1.2)	<0.001

Ch/Ad: Foods marketed to children or adolescents category; Non-Ch/Ad: foods not marketed to children or adolescents category; IR: Interquartile Range; n: number of the foods that had the correct amount of nutrient or energy; *p*-value: obtained for the Mann Whitney U test; ND: Not Determined due to the few foods declared with this nutrient content.

**Table 5:** Energy, protein, carbohydrates and sugar content in 14 food groups analysed according to two categories (Ch/Ad and non-Ch/Ad).

Energy and Nutrients in 100 g or 100 mL	Energy (kcal)			Protein (g)			Carbohydrates (g)			Sugar (g)		
	n	Median (IR)	*p*-Value	n	Median (IR)	*p*-Value	n	Median (IR)	*p*-Value	n	Median (IR)	*p*-Value
G1—Biscuits, breakfast cereals and cereal bars												
Ch/Ad	104	476 (460; 500)	<0.001	105	6.0 (5.3; 6.5)	<0.001	105	68 (66; 72)	0.077	104	29 (23; 35)	<0.001
Non-Ch/Ad	212	431 (378; 456)		212	7.7 (6.1; 9)		212	66 (62; 73.1)		208	18.2 (2.3; 23)	
G3—Canned, processed and derivatives vegetables												
Ch/Ad	25	39 (26; 69)	0.35	25	1 (0.7; 2.5)	0.632	25	4.7 (3.9; 10.8)	0.396	25	2.3 (1; 5.2)	0.957
Non-Ch/Ad	160	33 (20; 68)		160	1.2 (0.8; 1.8)		160	4.7 (2.3; 8.1)		148	2.5 (0.6; 5.5)	
G6—Cheese												
Ch/Ad	33	208 (165; 273)	<0.001	33	11 (10.5; 14)	<0.001	33	3.5 (2.5; 4.5)	<0.001	33	3.4 (1; 4.5)	<0.001
Non-Ch/Ad	131	333 (269; 387)		131	20 (14.5; 25)		130	1.5 (0.5; 3)		109	1 (0; 2.5)	
G7—Derivatives and processed fish/seafood												
Ch/Ad	52	198 (139; 243)	0.128	52	24 (20; 26)	<0.001	41	0 (0; 0.5)	0.002	41	0 (0; 0)	<0.001
Non-Ch/Ad	199	186 (145; 210)		204	21 (15.6; 23)		193	1 (0; 3.3)		190	0 (0; 0.9)	
G9—Fermented milk and dairy desserts												
Ch/Ad	35	86 (80; 113)	0.523	35	3.4 (3.1; 3.7)	<0.001	35	12.7 (11.4; 18.4)	0.839	34	12.6 (11.4; 16.2)	0.783
Non-Ch/Ad	117	97 (54; 112)		118	3.9 (3.5; 4.3)		117	13.5 (7.1; 17.1)		112	12.8 (6.1; 15.8)	
G12—Fruit beverages and juices												
Ch/Ad	33	49 (44; 58)	<0.001	33	0.6 (0.5; 0.8)	<0.001	33	11.4 (10.2; 13.7)	<0.001	33	11.3 (9.9; 13.7)	<0.001
Non-Ch/Ad	74	45 (25; 49)		73	0.2 (0; 0.3)		74	10 (5.9; 11.8)		72	9.9 (5.5; 11.6)	
G14—Milk and dairy beverages												
Ch/Ad	16	62 (58; 66)	<0.001	16	3 (2.8; 3.1)	0.003	16	9.5 (9; 10.3)	<0.001	16	9.5 (8.9; 10.3)	<0.001
Non-Ch/Ad	70	46 (37; 53)		70	3.1 (3.1; 3.2)		69	4.8 (4.7; 4.9)		67	4.8 (4.6; 4.9)	
G16—Meat												
Ch/Ad	28	229 (208; 245)	0.189	27	12 (11; 13)	<0.001	28	3.8 (1; 5.1)	0.272	20	0.5 (0.5; 1)	0.012
Non-Ch/Ad	219	185 (102; 312)		234	16 (13; 21.2)		232	2 (1; 4.5)		124	1.0 (0.5; 1.6)	
G19—Pastries and cakes												
Ch/Ad	27	442 (405; 462)	0.924	26	5.4 (4.6; 5.8)	0.833	27	54 (51; 58)	0.016	24	31.6 (27; 38.3)	0.090
Non-Ch/Ad	47	431 (399; 471)		46	5.5 (4.4; 6)		47	51 (49; 56)		46	28.6 (23; 35)	
G20—Processed Nuts and seeds												
Ch/Ad	15	598 (575; 624)	0.484	15	27.5 (24; 30)	0.011	15	10 (6.3; 12.9)	0.436	15	3.6 (2.5; 4.2)	0.0453
Non-Ch/Ad	33	612 (577; 632)		33	23 (19; 26)		33	10 (5.9; 24)		33	4.7 (2.9; 6)	
G22—Sauces												
Ch/Ad	15	131 (100; 203)	0.011	15	1.1 (0.9; 1.7)	0.785	14	23 (17; 31)	ND	11	19 (8.4; 25.4)	ND
Non-Ch/Ad	69	291 (139; 594)		68	1.1 (0.9; 21.7)		69	7 (3; 9.5)		63	3.7 (1.5; 6.2)	
G23—Snacks												
Ch/Ad	56	506 (469; 531)	0.538	56	6.2 (5.1; 6.9)	0.175	56	58 (54.5; 64)	0.008	56	2.4 (1.4; 3.8)	0.001
Non-Ch/Ad	37	519 (462; 532)		37	5.9 (4.8; 6.4)		37	51.8 (49; 59)		37	1.2 (0.6; 2.1)	
G24—Soft drinks												
Ch/Ad	28	25 (8; 39)	<0.001	28	0 (0; 0)	<0.001	28	5.9 (1.6; 9.4)	0.002	28	5.8 (1.6; 9.2)	<0.001
Non-Ch/Ad	57	22 (2; 37)		57	0 (0; 0)		56	5.1 (0.1; 8.8)		57	5 (0; 8.8)	
G25—Sweets and chocolates												
Ch/Ad	66	545 (398; 555)	0.01	65	5.5 (2.6; 7.3)	0.047	65	57 (53.5; 72.5)	<0.001	50	53.5 (41.4; 60.3)	<0.001
Non-Ch/Ad	205	546 (489; 571)		205	6.1 (4.8; 7.4)		205	50 (43; 54)		204	44 (34.8; 49)	

Ch/Ad: Foods marketed to children or adolescents; Non-Ch/Ad: foods not marketed to children or adolescents; IR: Interquartile range; *p*-value: obtained for the Mann Whitney U test; ND: Not determined due to the few foods declared with this nutrientcontent in at least one category.

**Table 6:** Total fat, saturated fat, fibre and salt content in 14 food groups analysed according to two categories (Ch/Ad and non-Ch/Ad).

Energy and Nutrients in 100 g or 100 mL	Total Fat (g)			Saturated Fat (g)			Fibre (g)			Salt (g)		
	n	Median (IR)	*p*-Value	n	Median (IR)	*p*-Value	n	Median (IR)	*p*-Value	n	Median (IR)	*p*-Value
G1—Biscuits, breakfast cereals and cereal bars												
Ch/Ad	105	20 (16; 23)	<0.001	104	8 (2.6; 13)	<0.001	64	2.7 (2.3; 3.9)	<0.001	103	0.6 (0.4; 1)	0.137
Non-Ch/Ad	212	14 (6.3; 18)		207	2 (1.3; 5)		195	5.5 (3.5; 9)		207	0.7 (0.3; 0.9)	
G3—Canned, processed and derivatives vegetables												
Ch/Ad	25	1 (0.6; 1.9)	0.006	23	0.3 (0.2; 0.5)	<0.001	25	1.2 (0.5; 2.5)	0.401	25	0.69 (0.5; 0.8)	0.461
Non-Ch/Ad	159	0.3 (0; 1.1)		147	0 (0; 0.1)		143	1.5 (1.1; 2.2)		155	0.7 (0.2; 0.93)	
G6—Cheese												
Ch/Ad	33	14 (10; 22)	<0.001	33	9.6 (7.4; 15)	<0.001	1	1 (1;1)	ND	33	1 (0.7; 1.6)	0.005
Non-Ch/Ad	129	28 (21; 32)		110	18.7 (14.3; 22.3)		10	0.3 (0.1;0.5)		112	1.5 (1.1; 2)	
G7—Derivatives and processed fish/seafood												
Ch/Ad	51	10 (4.9; 16)	0.84	51	1.5 (1.1; 2.7)	0.332	6	0 (0; 0)	ND	51	1.2 (1; 1.5)	0.002
Non-Ch/Ad	204	10 (7; 13)		203	2 (1.3; 2.5)		55	0 (0; 0.3)		193	1.5 (1; 1.6)	
G9—Fermented milk and dairy desserts												
Ch/Ad	35	2.6 (2; 3.2)	0.330	34	1.7 (1.3; 2)	0.263	15	0 (0; 0.2)	0.008	34	0.14 (0.1; 0.2)	0.011
Non-Ch/Ad	118	2.5 (0.2; 3.4)		113	1.6 (0.1; 2.2)		57	0.2 (0.1; 1)		112	0.1 (0.1; 0.1)	
G12—Fruit beverages and juices												
Ch/Ad	32	0.1 (0.1; 0.1)	<0.001	32	0 (0; 0)	0.896	26	0.7 (0.3; 1)	ND	33	0.002 (0.001; 0.002)	<0.001
Non-Ch/Ad	72	0 (0; 0)		72	0 (0; 0)		12	0.4 (0.1; 0.5)		71	0.01 (0; 0.03)	
G14—Milk and dairy beverages												
Ch/Ad	16	1.2 (1; 1.5)	0.173	16	0.8 (0.7; 1)	0.751	2	0.2 (0.2; 0.3)	ND	16	0.15 (0.14; 0.2)	0.068
Non-Ch/Ad	70	1.6 (0.5; 2.3)		66	0.9 (0.3; 1.1)		17	0 (0; 0)		67	0.13 (0.1; 0.2)	
G16—Meat												
Ch/Ad	28	18 (16; 20)	0.067	20	6.0 (5.1; 7)	1.22	0	--	ND	28	2 (1.8; 2.1)	0.018
Non-Ch/Ad	232	12 (2.5; 25.6)		134	4.5 (1.0; 8.9)		2	0 (0; 0)		226	2.1 (1.9; 3)	
G19—Pastries and cakes												
Ch/Ad	27	23 (16.5; 23)	0.715	24	7.5 (4.63; 12.3)	0.287	16	1.6 (1.4; 2.2)	0.016	25	0.5 (0.3; 0.8)	0.323
Non-Ch/Ad	48	23 (16.8; 23)		47	10.0 (5.9; 13.1)		43	2.3 (1.8; 3.2)		47	0.6 (0.5; 0.8)	
G20—Processed Nuts and seeds												
Ch/Ad	15	49.7 (45.5; 51.4)	0.969	15	5.5 (5.1; 5.8)	<0.001	15	8.6 (6.3; 9.1)	0.2873	15	2.2 (0.53; 3.8)	0.059
Non-Ch/Ad	33	49.7 (43; 54)		33	7.1 (5.6; 8.9)		25	7.6 (5.1; 8.3)		32	0.72 (0.4; 1.2)	
G22—Sauces												
Ch/Ad	15	0.5 (0.1; 11.4)	<0.001	11	0 (0; 1.8)	ND	6	0.9 (0.7;1.4)	ND	8	2.1 (1.8; 2.9)	ND
Non-Ch/Ad	69	26.5 (6.9; 61.8)		61	4.4 (1.2; 9.5)		43	0.3 (0.1;1.3)		54	1.5 (1.2; 2)	
G23—Snacks												
Ch/Ad	56	26 (21.8; 33)	0.104	56	4.4 (3.1; 8)	0.01	56	3.3 (2; 4.4)	0.309	53	1.5 (1; 2))	0.549
Non-Ch/Ad	37	31 (20.3; 34)		37	3.4 (2.7; 3.9)		37	3.9 (2.6;4.9)		37	1.7 (1; 2.4)	
G24—Soft drinks												
Ch/Ad	28	0 (0; 0)	0.014	28	0 (0; 0)	NA	7	0 (0; 0)	ND	28	0.03 (0; 0.1)	0.895
Non-Ch/Ad	56	0 (0; 0)		56	0 (0; 0)		4	0.1 (0; 0.3)		57	0.03 (0.01; 0.1)	
G25—Sweets and chocolates												
Ch/Ad	65	31.6 (3.5; 34)	<0.001	50	5.4 (1.7; 16.9)	<0.001	20	1.3 (0; 2.8)	0.221	51	0.17 (0.1; 0.3)	0.075
Non-Ch/Ad	205	35 (26.1; 40)		205	19 (15; 22)		28	1.6 (0.3; 7.2)		205	0.13 (0.1; 0.2)	

Ch/Ad: Foods marketed to children or adolescents; Non-Ch/Ad: foods not marketed to children or adolescents; IR: Interquartile range; *p*-value: obtained for the Mann Whitney U test; NA: Not Applicable; ND: Not Determined due to the few foods declared with this nutrient content in at least one category.
